# Cystic adventitial disease of the popliteal artery: an infrequent cause of intermittent claudication

**DOI:** 10.1590/S1679-45082014RC2818

**Published:** 2014

**Authors:** Paulo Kauffman, Sergio Kuzniec, Roberto Sacilotto, Marcelo Passos Teivelis, Nelson Wolosker, Adriano Tachibana

**Affiliations:** 1 Hospital Israelita Albert Einstein, São Paulo, SP, Brazil.; 2 Instituto de Assistência Médica ao Servidor Público Estadual “Francisco Morato de Oliveira”, São Paulo, SP, Brazil.

**Keywords:** Adventitia, Diagnosis, differential, Intermittent claudication, Popliteal artery, Case reports

## Abstract

Intermittent claudication is frequently associated with atherosclerotic disease, but differential diagnosis must be sought in patients with no traditional risk factors. Cystic adventitial disease, of unknown etiology, most frequently affects the popliteal artery, and occasionally presents as intermittent claudication. We report a case of this disease and the surgical treatment, and discuss some aspects related to etiopathogenesis, diagnosis and treatment of this condition.

## INTRODUCTION

Cystic adventitial disease of the popliteal artery is a rare clinical entity of unknown etiology, which may present as calf intermittent claudication, usually unilateral, in healthy middle-aged individuals, who do not smoke and have no risk factors for atherosclerotic disease.

The objective of the current report is to present a case of intermittent claudication caused by cystic adventitial disease of the popliteal artery, and discuss its etiology, diagnosis and treatment.

## CASE REPORT

A 64-year-old male patient, business administrator, from São Paulo (SP), played tennis 30 days before the medical appointment and complained of pain in the posterior muscles of the left leg, which was relieved after resting. Whenever he played tennis or walked fast, pain would recur. He did not smoke and had no diabetes or dyslipidemia. He was on irregular use of enalapril maleate for hypertension. Upon physical examination, he was moderately hypertensive (blood pressure 150/90mmHg). The arterial examination revealed decreased intensity in the popliteal and podal pulses in the left lower limb. Computed tomography angiography (CTA) showed dilated left popliteal artery, with irregularities and stenosis of the lumen, anechoic content in the wall, which the radiologist interpreted as aneurysm with parietal thrombi. Duplex-scan demonstrated that the anechoic images in the wall were septated adventitial cysts with significant stenosis of the arterial lumen (70%).

**Figure 1:**
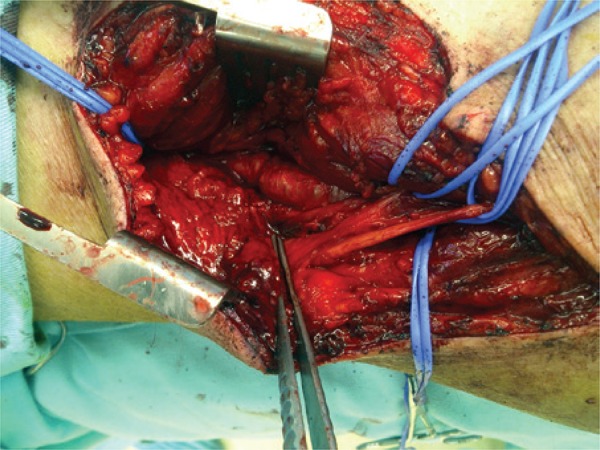
Surgical site. Observe parietal irregularities in the popliteal artery

**Figure 2:**
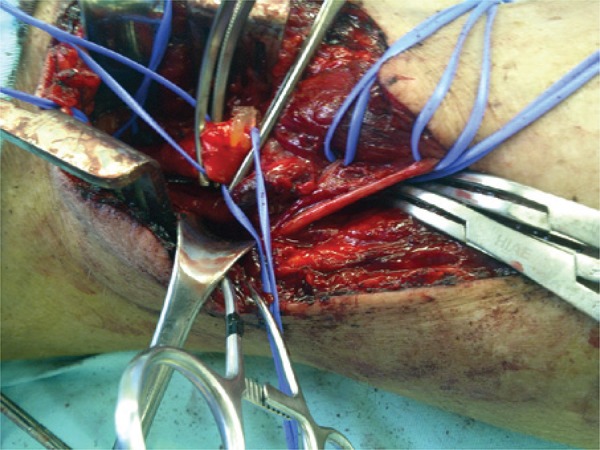
Incision in the artery. Drainage of a viscous material

**Figure 3:**
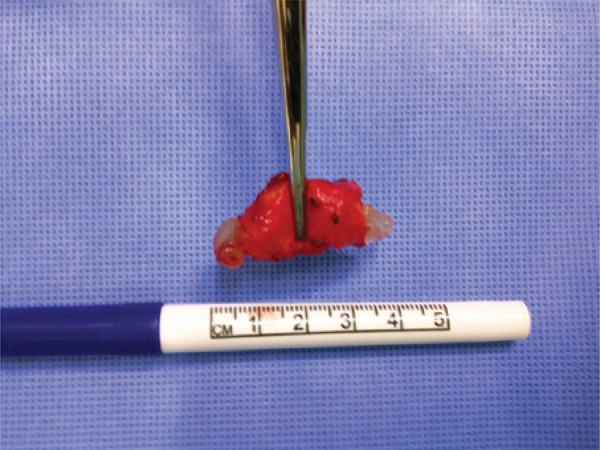
Surgical specimen

The patient was operated on by direct approach of the affected popliteal artery, with partial resection and replacement by an autogenous great saphenous vein graft. The artery was dilated with increased texture and parietal irregularities (Figure 1). After excision, a yellowish viscous material was observed in its adventitia (Figures 2 and 3). The pathological examination revealed cystic adventitial disease of the popliteal artery.

## DISCUSSION

The cystic adventitial disease was described in 1947 in a man presenting intermittent claudication due to a myxomatous tumor in the external iliac artery.^(1)^ It predominates in males in the ratio of 15:1, and it is more frequent in the fourth and fifth decades of life. It is estimated to occur in 1 out of every 1,200 of individuals suffering from claudication. It is characterized by formation of multiple cysts filled with a clear or yellowish viscous material, rich in mucoproteins and mucopolysaccharides, located in the adventitia and compressing the lumen of the vessel, which may lead to obstruction. Hence, it is different from the cystic degeneration of the medial layer that is observed in the aorta and in the elastic or muscular arteries, when the smooth muscles in this layer are affected.

The cystic adventitial disease (or cystic adventitial degeneration) predominantly occur in arteries, and rarely in veins and the popliteal artery is the most often involved (85 to 90% of cases). Regarding histopathology, there are various cysts with viscous material containing proteoglycans, mucopolysaccharides and mucoproteins, in addition to hyaluronic acid, located in the adventitia of the vessel.^(2)^

The etiopathogenesis remains controversial, and there are many theories trying to explain the formation of these cysts. One approach is that these vessels are close to joints, and this may be related to the theory that repeated microtraumas cause cystic degeneration of the adventitia, due to stretching and distortion of the arterial segment adjacent to the joint.^(3)^ However, this disease is not frequent in athletes. There is an articular (synovial) theory that one branch (the middle genicular artery) would be the conduit taking synovial cells from the knee joint, which would dissect the adventitia and implant in the popliteal artery. The embryological theory would explain the disease by implanting mesenchymal cells of adjacent joints in the adventitia of blood vessels during the embryological development.^(4)^

Due to similar symptoms, the differential diagnosis of the cystic adventitial disease of the popliteal artery should be made with peripheral arterial disease and popliteal entrapment syndrome. Due to muscle activity and flexion of the knee, the cysts may compress the artery, explaining pain reported by our patient. The arterial pulses can be normal in the affected limb in the neutral position, and they are less intense or disappear with knee flexion. In the present case, the popliteal and podal pulses in the affected leg were weaker as compared to the contralateral limb, due to a stenotic arterial lesion visualized in the computed tomography angiography.

Ultrasonography is a non-invasive method, with greater sensitivity to demonstrate arterial cystic disease:^(5)^ in a gray scale (B-mode), it shows the presence of cysts as hypoechoic or anechoic masses, involving the vessel; on Doppler, stenosis or arterial occlusion are observed. In the case described, this imaging method was crucial to make diagnosis of the disease. Other imaging methods have also been useful to diagnose arterial cystic disease, such as computed tomography (CTA) and magnetic resonance imaging angiography (MRA). In our patient, CTA was not enough to elucidate diagnosis, since it suggested a popliteal artery aneurysm. Neverthless, the irregularities and stenosis of the vessel lumen – which are not found in aneurysms, led to uncertain diagnosis. Hence, a more accurate analysis of tomography after ultrasound led to the correct diagnosis of cystic disease.

Since this condition is rare, there is no consensus about treatment. Although there are occasional reports on spontaneous resolution of the disease, mainly when the arterial wall cysts have some communication with the knee joint,^(6)^ early treatment can prevent thrombosis of the popliteal artery. The therapy most frequently used for this disease is the resection of the impaired arterial segment and replacement by an autogenous venous graft,^(2)^ which was performed in the present case. This management was chosen due to extension of the disease in the arterial wall, associated to marked stenosis of the lumen. Several authors preserve the artery by just excising the adventitial cyst, but recurrence of the disease is not uncommon.^(7)^ Ultrasound-guided aspiration of the cyst content has been described as a less invasive method.^(8)^ Needle aspiration is not always possible since the material is quite thick. Endovascular treatment has been frequently employed today in most cases of peripheral arterial disease,^(9)^ but this is not true for this condition, since it has not been effective,^(10)^ probably because the popliteal mobile segment is usually involved.

Cystic adventitial disease of the popliteal artery should be considered in the differential diagnosis of intermittent claudication, especially in patients with no traditional risk factors for atherosclerosis.
