# Supplementary material for: Cystic adventitial disease of the popliteal artery: an infrequent cause of intermittent claudication
Source: Einstein (Sao Paulo). 2014 Jul-Sep;12(3):358–60. doi: 10.1590/S1679-45082014RC2818 (PMC4872951; doi:10.1590/S1679-45082014RC2818)
Supplement: Translation

# Translation: Doença cística adventicial da artéria poplítea: causa infrequente de claudicação intermitente

**Authors:** Paulo Kauffman, Sergio Kuzniec, Roberto Sacilotto, Marcelo Passos Teivelis, Nelson Wolosker, Adriano Tachibana

**Affiliations:** 1 Hospital Israelita Albert Einstein, São Paulo, SP, Brasil.; 2 Instituto de Assistência Médica ao Servidor Público Estadual “Francisco Morato de Oliveira”, São Paulo, SP, Brasil.

**Keywords:** Túnica adventícia, Diagnóstico diferencial, Claudicação intermitente, Artéria poplítea, Relatos de casos

## Abstract

A claudicação intermitente está frequentemente associada à doença aterosclerótica, mas diagnósticos diferenciais devem ser pesquisados em pacientes sem fatores de risco tradicionais. A doença cística adventicial, de etiologia incerta, acomete em maior proporção a artéria poplítea e, eventualmente, apresenta-se como claudicação intermitente. Apresentamos um caso da doença e seu manejo cirúrgico, e discutimos a etiopatogenia, os aspectos diagnósticos e terapêuticos da enfermidade.

## INTRODUÇÃO

A doença cística adventicial da artéria poplítea é uma entidade clínica pouco frequente, cuja etiologia é desconhecida, que pode se manifestar por dor do tipo claudicação intermitente de panturrilha, geralmente unilateral, em indivíduos saudáveis de meia-idade, não fumantes e sem fatores de risco para doença aterosclerótica.

O objetivo do presente trabalho foi apresentar um caso de claudicação intermitente cuja causa era a doença cística adventicial da artéria poplítea, discutindo sua etiologia, diagnóstico e tratamento.

## RELATO DO CASO

Paciente ASP, masculino, 64 anos, administrador, procedente de São Paulo (SP), há 30 dias, jogando tênis, referiu dor na musculatura posterior da perna esquerda, que cedeu com o repouso. A dor passou a ocorrer sempre que jogava tênis ou andava depressa. Negava tabagismo, diabete, dislipidemia. Referia uso inconsistente de maleato de enalapril para hipertensão. No exame físico, estava moderadamente hipertenso (pressão arterial de 150/90mmHg). O exame arterial revelou diminuição de intensidade dos pulsos poplíteo e podais no membro inferior esquerdo. Angiotomografia revelou artéria poplítea esquerda dilatada, com irregularidades e estenose da luz, além de conteúdo anecoico em sua parede, interpretada pelo radiologista como aneurisma com trombos parietais. Duplex-scan mostrou que as imagens anecoicas na parede eram cistos septados adventiciais com estenose significativa da luz arterial (70%).

O paciente foi submetido à cirurgia: abordagem direta da artéria poplítea comprometida, com ressecção parcial e substituição por enxerto venoso (veia safena magna autógena). A artéria apresentava-se dilatada, com consistência aumentada e irregularidades parietais (Figura 1). Após secção, observou-se saída de material gelatinoso amarelado de sua adventícia (Figuras 2 e 3). O exame anatomopatológico confirmou tratar-se de doença cística adventicial da artéria poplítea.

**Figura 1:** Sítio operatório. Observar irregularidades parietais da artéria poplítea

**Figura 2:** Secção da artéria. Saída de material gelatinoso

**Figura 3:** Espécime cirúrgico

## DISCUSSÃO

A doença cística adventicial foi descrita em 1947 em um homem que apresentava claudicação intermitente como um tumor mixomatoso na artéria ilíaca externa.^(1)^ Predomina no gênero masculino na proporção de 15:1, incidindo com maior frequência na quarta e quinta décadas de vida e em 1:1.200 dos claudicantes. Caracteriza-se pela formação de múltiplos cistos preenchidos por material gelatinoso rico em mucoproteínas e mucopolissacárides, incolor ou amarelado, localizados na camada adventicial, que comprimem a luz do vaso, podendo resultar em sua obstrução. Difere, assim, da degeneração cística da média, que ocorre na aorta e nas artérias elásticas ou musculares, na qual a musculatura lisa dessa camada fica comprometida.

A doença cística adventicial (ou degeneração cística adventicial) acomete predominantemente artérias e raramente as veias. Dentre as artérias, a poplítea é a mais frequentemente acometida (85 a 90% dos casos). Do ponto de vista histopatológico, encontram-se vários cistos com material mucinoso contendo proteoglicanos, mucopolissacárides e mucoproteínas, além de ácido hialurônico, localizados na adventícia do vaso.^(2)^

A etiopatogenia permanece controversa, havendo várias teorias que tentam explicar a formação desses cistos. A proximidade desses vasos com as articulações fez surgir a teoria dos microtraumas repetidos com degeneração cística da adventícia, devido ao alongamento e à distorção do segmento arterial adjacente à articulação.^(3)^ No entanto, não se observa ocorrência mais frequente dessa doença em atletas. Outra teoria é a articular (sinovial), segundo a qual um ramo (artéria genicular média) seria o conduto que levaria células sinoviais da articulação do joelho que dissecariam a adventícia e se implantariam na artéria poplítea. A teoria embrionária explicaria a doença pela implantação de células mesenquimais de articulações adjacentes na adventícia de vasos sanguíneos durante o desenvolvimento embrionário.^(4)^

O diagnóstico da doença cística adventicial da artéria poplítea se confunde em razão da similaridade dos sintomas com os portadores de doença obstrutiva crônica periférica e com a síndrome do encarceramento da poplítea. Com a atividade muscular e a flexão do joelho, pode haver compressão da artéria pelos cistos, o que explicaria a queixa do paciente. Os pulsos arteriais podem ser normais no membro afetado na posição neutra, diminuindo de intensidade ou desaparecendo com a flexão do joelho. No caso em questão, os pulsos poplíteo e podais no membro afetado estavam mais fracos do que no membro contralateral, em decorrência da lesão arterial estenosante visualizada na angiotomografia.

O ultrassom é um método não invasivo e de maior sensibilidade para demonstrar a doença cística arterial:^(5)^ em escala cinza (Modo B), revela a presença dos cistos como massas hipo ou anecoicas, envolvendo o vaso e, no modo Doppler, mostra a estenose ou oclusão arterial. No caso descrito, esse método de imagem foi decisivo para o diagnóstico da doença. Outros métodos de imagem também têm sido úteis no diagnóstico da doença cística arterial, como a tomografia computadorizada, a angiotomografia e a angiorressonância. A angiotomografia, no nosso paciente, não foi suficiente para elucidar o diagnóstico, pois sugeriu tratar-se de aneurisma da artéria poplítea. No entanto, as irregularidades e a estenose da luz do vaso, achados não encontrados nos casos de aneurisma, deixaram em dúvida esse diagnóstico; assim, análise mais acurada da imagem tomográfica, após se ter a avaliação ultrassonográfica, permitiu o diagnóstico correto da doença cística.

Devido à raridade dessa condição, não existe consenso sobre o tratamento. Embora haja descrição ocasional de resolução espontânea da doença, principalmente quando os cistos da parede arterial mostram comunicação com a articulação do joelho,^(6)^ o tratamento precoce da afecção pode prevenir trombose da artéria poplítea. Os métodos terapêuticos mais frequentemente utilizados são a ressecção do segmento arterial afetado e a substituição por enxerto venoso autógeno,^(2)^ técnica empregada no presente caso. Essa conduta foi adotada devido à extensão da doença na parede arterial associada à estenose importante da luz do vaso. Preservação da artéria com somente excisão do cisto adventicial foi empregada por vários autores, porém não é infrequente a recidiva da doença.^(7)^ A aspiração do conteúdo do cisto guiada por ultrassonografia tem sido descrita como um método terapêutico menos invasivo.^(8)^ Como se trata de material espesso, nem sempre é possível sua aspiração com agulha. O tratamento endovascular, aplicado com grande frequência nos dias de hoje para a maior parte dos casos de doença arterial obstrutiva periférica,^(9)^ tem sido relatado com pouca frequência e não tem se mostrado efetivo nessa afecção,^(10)^ − talvez porque o segmento móvel da artéria poplítea é usualmente o mais acometido.

Trata-se de doença que deve ser considerada no diagnóstico diferencial de claudicação intermitente, em especial em pacientes com ausência de fatores de risco tradicionais para aterosclerose.
